# Sensation seeking in 3- to 6-year-old children: associations with socio-demographic parameters and behavioural difficulties

**DOI:** 10.1186/s12887-019-1450-6

**Published:** 2019-03-11

**Authors:** Myriam Haas, Andreas Hiemisch, Mandy Vogel, Oleg Wagner, Wieland Kiess, Tanja Poulain

**Affiliations:** 1 LIFE Leipzig Research Center for Civilization Diseases, University of Leipzig, Philipp-Rosenthal-Strasse 27, 04103 Leipzig, Germany; 2 Department of Women and Child Health, Hospital for Children and Adolescents and Center for Pediatric Research (CPL), Leipzig University, Liebigstrasse 20a, 04103 Leipzig, Germany

**Keywords:** Sensation seeking, Socio-economic status, Behavioural difficulties, Children

## Abstract

**Background:**

The present study investigates whether sensation seeking in pre-school-age children is associated with age, gender and socio-economic status, and how sensation seeking relates to behavioural difficulties.

**Methods:**

The study sample consisted of 423 three- to six-year-old children whose parents had completed questionnaires on the children’s sensation seeking (Sensation Seeking Scale for Young Children), socio-economic status, and behavioural difficulties (Strengths and Difficulties Questionnaire). Multiple linear regression models were applied to investigate associations between sensation seeking and age, gender, socio-economic status and behavioural difficulties.

**Results:**

Sensation seeking was significantly higher in male vs. female children but not associated with children’s socio-economic status. Furthermore, sensation seeking was positively correlated with conduct problems, but negatively with emotional symptoms and peer-relationship problems.

**Conclusion:**

These results replicate findings in adults and strengthen the assumption that sensation seeking is a personality trait that is already observable in early childhood. Furthermore, our results suggest relationships between higher levels of sensation seeking and externalising behaviour as well as relationships between lower levels of sensation seeking and internalising behaviour.

**Trial registration:**

LIFE Child study: ClinicalTrials.gov, clinical trial number NCT02550236.

## Background

Sensation seeking is a personality trait defined by the seeking of novel and intense sensations and experiences, and the willingness to take risks for the sake of such experience [1]. High sensation seekers become bored if stimuli and experiences become repetitive [2]. A positive outcome of high sensation seeking seems to be the ability to better manage stress and adversity [3].

Sensation seeking has been widely studied in adults [2, 4–14] and significant correlations have been found between risky behaviours, such as smoking and drug use, and aggressive behaviour. In contrast, only a little is known about sensation seeking in young children [15–19]. Studies have shown that high sensation seeking in children and preadolescents is associated with greater vulnerability to unintentional injuries, substance use, rule-breaking behaviour and aggressive behavioural disorders [18, 20–23]. Furthermore, sensation seeking children have been shown to exhibit a greater propensity for risk-taking play behaviour [24], including being more likely to play violent video games [23].

The present study is the first, to our knowledge, that has investigated associations between sensation seeking and age, gender, SES and behavioural problems in a sample of preschool children.

The primary aim of this study was to investigate the relationship between sensation seeking and age, gender, and the socio-economic status (SES). In adolescents and adults, age and gender have been shown to be related to sensation seeking traits [6, 9, 25]. With respect to gender, previous studies have reported higher sensation seeking in men versus women [1, 10, 12, 26, 27]. Similarly, boys (aged seven or older) were found to exhibit higher levels of sensation seeking than girls [23]. Concerning age differences, Zuckerman [28] postulated that sensation seeking is related to developmental phases. It is suggested that sensation seeking increases between childhood and adolescence, peaks between 16 and 19, and declines or stabilises in late adolescence or young adulthood [15, 16, 29–32]. However, previous studies have found no, or only small, associations between sensation seeking and SES (income, education, occupational status) in adolescents and adults [9, 12, 26].

Consequently, we expected to observe more sensation seeking in boys compared to girls and greater levels of sensation seeking in older versus younger preschool children, but no relation between sensation seeking and SES.

Our second objective was to investigate associations between sensation seeking and behavioural difficulties in children. To date, very few studies have examined this relationship. Previous findings suggest an association between sensation seeking and externalising behavioural difficulties, namely conduct problems [33, 34] in children and adolescents, and hyperactivity symptoms in adults [35]. In respect to internalising problems (e.g. emotional problems), previous findings are mixed. Whereas Xu and Ortin [36, 37] observed positive associations between sensation seeking and depressive symptoms, Giannoni-Pastor [38] did not find a link between the two, and Kövi [39] observed a higher prevalence of depression in adults who exhibited lower levels of sensation seeking. Based on these findings, we hypothesised that young children who show more signs of sensation seeking might have more behavioural difficulties than children who show fewer signs of sensation seeking, especially with respect to externalising behaviours.

## Methods

### The LIFE child study

The data analysed in the present study were collected as part of the LIFE Child study, a large childhood cohort study conducted in Leipzig, Germany. The LIFE Child study aims to monitor healthy children from birth to adulthood to understand the development of lifestyle diseases such as diabetes, obesity, and mental disorders [40]. Depending on the children’s age, the comprehensive study program contains different medical, psychological and sociodemographic assessments as well as the collection of biological samples. The recruitment of study participants is based on a collaborative network of hospitals, public health centres, nursery schools, and schools [41]. More than 4000 children and adolescents aged between 0 and 18, and their parents, have participated since the study was initiated in 2011. The study was approved by the Ethics Committees of the Medical Faculty of the University of Leipzig, Germany (Reg. No. 264–10-19,042,010).

### Participants

The study sample consisted of 432 children aged between 3 and 6 who had participated in the LIFE Child study between 2012 and 2016. For each participating family, data were only included for one, randomly selected child. Nine children had to be excluded due to missing data. Thus, the final sample consisted of 423 children (51.3% male, 48.7% female, mean age = 4.7 years, range 3.5–6.5 years). Informed parental consent was provided in writing for all child participants.

### Measurements

#### Sensation seeking scale for young children (SSSYC)

Sensation seeking was assessed using the adapted German version of the Sensation Seeking Scale for Young Children (SSSYC) [18]. This questionnaire includes 24 items in the three subcategories ‘novelty seeking’ (NS, 8 items), ‘behavioural intensity’ (BI, 6 items) and ‘thrill seeking’ (TS, 10 items). NS items measure aversion to repetition and the propensity to seek new experiences, e.g. ‘playing a game that he/she never played before’. BI items assess the desire to engage in activities involving speed or danger, e.g. ‘climbing a tree’. TS items measure the need for a variety of emotional challenges that raise excitement, e.g. ‘listening to loud, bouncy music’. For each item, parents were asked to choose which of two behaviours describes their child better, with one behaviour reflecting high sensation seeking (1 point), e.g. ‘Go down a slide fast headfirst’, and the other reflecting low sensation seeking (0 points), e.g. ‘Go down a slide feet first’. In the event that one answer was missing, the average score of the subscale was used in place of the missing value. In the event that more than one answer was missing in a single subscale, the child was excluded from the analyses. In the present sample, the internal consistency of the questionnaires’ overall score (Cronbach’s alpha) was 0.76.

#### Socio-economic status (SES)

The participants’ SES was represented by an index (so called WSI Stratification Index) that combines information on three main indicators of SES, namely equivalent household income, parental education level, and parental occupational status [42–44]. The information used to calculate these SES indicators was collected using a questionnaire that was completed by the parents. Based on their responses, each indicator was assigned a score ranging from a minimum of 1 to a maximum of 7 points [44]. All three indicators contributed equally to the resulting points score [44]. Therefore, the minimum score for the WSI Stratification Index was 3, and the maximum score was 21. Based on this score, each family could be assigned a ‘lower’ (score between 3 and 8.40), ‘middle’ (score between 8.5 and 15.4), or ‘higher’ SES (score between 15.5 and 21) [44]. 412 parents (97.4%) provided complete information on their SES. In the present sample, 9.0% (*N* = 37) of the study participants belonged to the lower social milieu, 56.8% (*N* = 234) to the middle social milieu, and 34.2% (*N* = 141) to the higher social milieu.

#### Strengths and difficulties questionnaire (SDQ)

Behavioural problems were assessed using the parent report questionnaire from the German-language version of the SDQ [45, 46]. This standard screening instrument contains 25 items in 5 ‘scales’. The scale ‘pro-social behaviour’ is assessed as a psychological strength. In contrast, the other 4 scales, “hyperactivity/inattention’ (e.g. restlessness), ‘emotional symptoms’ (e.g. having many worries), ‘peer relationship problems’ (e.g. being picked on or bullied) and ‘conduct problems’ (e.g. often fighting with other children) are concerned with behavioural difficulties. The scores on the four problem scales are added together to produce an overall difficulties score. For each item, parents were asked to choose one of three answer categories (‘0 = not true’, ‘1 = somewhat true’ and ‘2 = certainly true’). In the normative sample, the German translation of the SDQ showed a good internal consistency (Cronbach’s Alpha) of 0.82 [46]. In the present study sample, the internal consistency (Cronbach’s Alpha) was 0.81 for the total difficulties score.

#### Statistical analyses

Descriptive statistics (mean and standard deviation) for the SSSYC are presented for the whole sample and differentiated by gender and age. The relationship between the scores on the different SSSYC scales was estimated by calculating Pearson correlations.

Levels of association between sensation seeking and parameters of age, gender, and SES were estimated separately using multiple linear regressions with age, gender and SES (as a continuous measure ranging from 3 to 21) as independent variables and either the overall SSSYC score or the three single scale values NS, BI, TS as dependent variables. Interaction effects between SES and age/gender were assessed using a three factorial analysis of variance, with the overall SSSYC score as the dependent variable and age (4 years vs. 5 years vs. 6 years), gender (male vs. female), and SES (low vs. middle vs. high) as factorial independent variables. Possible relationships between sensation seeking and behaviour difficulties were assessed using multiple linear regressions with the SDQ single scale values as independent variables and the overall SSSYC score or the single scale values of the SSSYC as dependent variables. These associations were adjusted for age, gender, and SES. All statistical models were checked for interactions between the independent variables and age or gender.

## Results

### Sensation seeking in the present sample

The mean and standard deviation values for the different SSSYC subscales are shown in Table 1. The average overall score ranged from 10.51 (SD 4.34) for girls (3.5–4.4 years in age) to 13.54 (SD 3.64) for boys (5.5–6.5 years in age).

**Table 1:** Sensation Seeking Scale for Young Children (SSSYC): mean (M) and standard deviation (SD) values by age and gender for *N* = 423 children aged 3 to 6

		SSSYC overall			NS		BI		TS	
Age	Gender	N	M	SD	M	SD	M	SD	M	SD
all	male	217	12.52	(4.06)	3.52	(1.86)	2.98	(1.62)	6.03	(2.01)
	female	206	11.24	(4.26)	3.56	(1.82)	2.84	(1.82)	4.84	(2.04)
3.5–4.4	male	111	11.85	(4.35)	3.30	(2.03)	2.85	(1.65)	5.70	(2.10)
	female	92	10.51	(4.34)	3.12	(1.94)	2.76	(1.82)	4.62	(2.05)
4.5–5.4	male	57	12.95	(3.62)	3.44	(1.59)	3.13	(1.54)	6.37	(1.87)
	female	51	11.47	(4.03)	3.78	(1.69)	2.73	(1.67)	4.96	(2.12)
5.5–6.5	male	49	13.54	(3.64)	4.11	(1.67)	3.07	(1.67)	6.36	(1.85)
	female	63	12.14	(4.20)	4.02	(1.61)	3.05	(1.95)	5.07	(1.95)

Age in years, *NS* Novelty Seeking, *BI* Behavioural Intensity, *TS* Thrill Seeking

The correlations between the scores on the different SSSYC scales are positive and highly significant (all *p* < .001). However, the associations between BI and TS appear to be much stronger (*r* = .615) than the associations between BI and NS (*r* = .173) or between TS and NS (*r* = .176).

### Associations between sensation seeking and parameters of age, gender, and SES

**Fig. 1:**
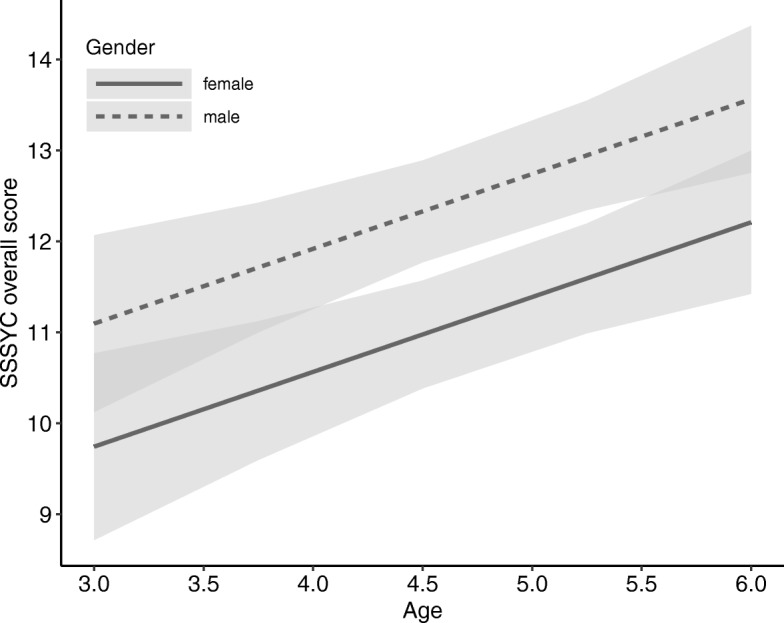
Gender-specific effect plot illustrating the estimated overall SSSYC score (+ confidence interval) depending on age. 3- to 6-year-old boys scored higher on the overall sensation seeking scale than girls

Table 2 presents the various associations identified between scores in the different SSSYC subscales and age, gender, and SES. With respect to gender, the analyses revealed significant gender differences in the overall SSSYC score (*β* = −.162, *b* = − 1.35, *p* = .001, see also Fig. 1). Male children were estimated to score on average 1.35 points higher than female children. Looking at the single SSSYC scales, however, only scores on the TS scale were related to gender (*β* = −.291, *b* = − 1.23, *p* < .001). No significant gender effects were observed in NS (*β* = .000, *b* = 0.00, *p* = .993) and BI (*β* = −.036, *b* = − 0.12, *p* = .464). These findings indicate that the boys were found to be more ‘thrill seeking’ than the girls, whereas levels of behavioural intensity and novelty seeking did not differ between boys and girls.

**Table 2:** Associations between scores on the different scales of the SSSYC and gender, age, and SES values

		*β*	*b*	*p*
SSYC Total	Gender	−.162	−1.35	.001
	Age	.169	0.82	.001
	SES	.026	0.03	.596
NS	Gender	.000	0.00	.993
	Age	.188	0.40	.000
	SES	.043	0.02	.383
BI	Gender	−.036	−0.12	.464
	Age	.069	0.13	.167
	SES	.006	0.00	.905
TS	Gender	−.291	−1.23	<.001
	Age	.116	0.28	.015
	SES	.009	0.00	.848

*NS* Novelty Seeking, *BI* Behavioural Intensity, *TS* Thrill Seeking, *β* = standardised regression coefficient, *b* = non-standardised regression coefficient, level of significance *p* < 0.05. Socio-economic status was not found to be related to sensation seeking in children. Associations were found between age and gender values and sensation seeking, whereby male and older children tend to score higher on the sensation seeking scale

In addition to the gender differences, the significant associations found between participant age and the overall SSSYC score (*β* = .169, *b* = 0.82, *p* < .001), the NS score (*β* = .188, *b* = 0.40, *p < .001*), and the TS score (*β* = .116, *b* = 0.28, *p* = .015) indicate that older children are more sensation seeking than younger children, especially with respect to thrill seeking and novelty seeking. For example, according to the statistical model, the overall SSSYC score increased by approximately 0.82 points per additional year of life. We found no significant age effect in BI (*β* = .069, *b* = 0.13, *p* = .167). Figure 1 illustrates the association between the overall SSSYC score and age as a function of gender.

As shown in Table 2, SES was not shown to be related to either the overall SSSYC score (*β* = .026, *b* = 0.03, *p* = .596) or to the subscales NS (*β* = .043, *b* = 0.02, *p* = .383), BI (*β* = .006, *b* = 0.00, *p* = .905) and TS (*β* = .009, *b* = 0.00, *p* = .848). Furthermore, we did not find a significant SES*age interaction (*F* = .620 (df = 4), *p* = .649) or a significant SES*gender interaction (*F* = .594 (df = 2), *p* = .552).

**Fig. 2:**
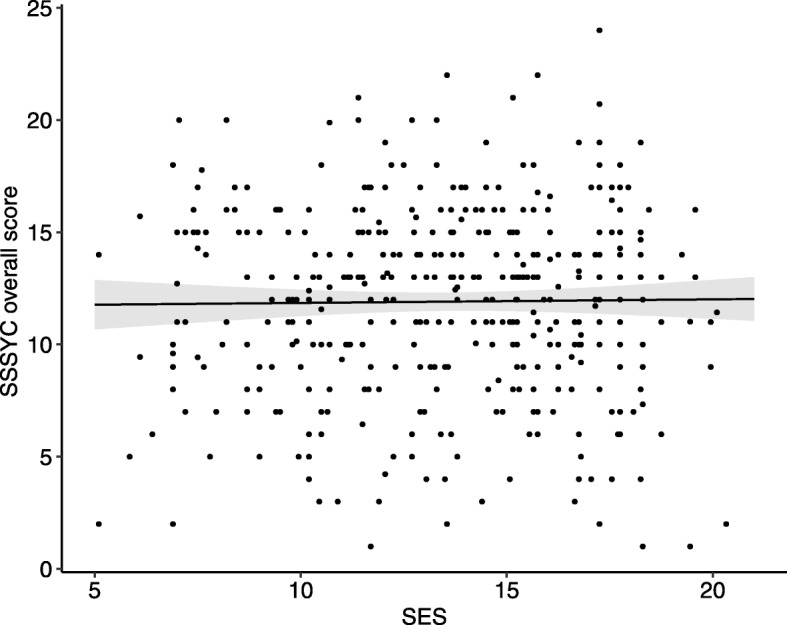
Scatterplot illustrating the independence of the overall SSSYC score in relation to SES. Sensation seeking and the socio-economic status were not related in our study sample

The findings show that sensation seeking and SES were not related in our study sample, although it should be noted that families with a low SES are underrepresented in this data (see Fig. 2).

### Associations between sensation seeking and behavioural difficulties

The associations between scores on the different subscales of the SSSYC and scores on the SDQ scales are shown in Table 3. The analyses revealed significant negative associations between the overall SSSYC score and both emotional problems (*β* = −.293, *b* = −.689, *p* < .001) and peer relationship problems (*β* = −.110, *b* = −.311, *p* = .031). For every one point increase on the ‘emotional problems’ scale, the overall SSSYC score drops by 0.689 points. On the other hand, we found positive relationships between the overall SSSYC score and conduct problems (*β* = .190, *b* = .534, *p* = .001). A one point increase on the ‘conduct problems’ scale sees the overall SSSYC score increase by 0.534 points. Only the association between the overall SSSYC score and hyperactivity/inattention was not significant (*β* = .077, *b* = .132, *p* = .169).

**Table 3:** Associations between scores on the different scales of the SSSYC and behavioural difficulties

	SDQ	Emotional symptoms	Conduct problems	Hyperactivity/inattention	Peer relationship problems	Pro-social behaviour
SSSYC Total	*β*	−.293	.190	.077	−.110	.073
	*b*	−.689	.534	.132	−.311	.195
	p	<.001	.001	.169	.031	.161
NS	*β*	−.120	.102	−.062	−.010	.094
	*b*	−.123	.125	−.047	−.012	.110
	*p*	.021	.092	.289	.854	.090
BI	*β*	−.251	.172	.103	−.193	.025
	*b*	−.240	.196	.072	−.223	.027
	*p*	<.001	.003	.072	<.001	.634
TS	*β*	−.275	.149	.123	−.053	.042
	*b*	−.325	.211	.107	−.075	.056
	*p*	<.001	.008	.026	.294	.412

*NS* Novelty Seeking, *BI* Behavioural Intensity, *TS* Thrill Seeking, *β* = standardised regression coefficient, *b* = non- standardised regression coefficient, level of significance *p* < 0.05. Higher sensation seeking is associated with conduct problems, whereas lower sensation seeking is related to emotional symptoms and peer-relationship problems

Looking at the constituent scales within the SSSYC, higher scores on the NS scale were significantly associated with fewer emotional problems (*β* = −.120, *b* = −.123, *p* = .021). Higher scores on the BI scale were associated with fewer emotional problems (*β* = −.251, *b* = −.240, *p* < 0.001), fewer peer relationship problems (*β* = −.193, *b* = −.223, *p* < .001), and more conduct problems (*β* = .172, *b* = .196, *p* = 0.003). Higher scores on the TS scale were significantly related to fewer emotional problems (*β* = −.275, *b* = −.325, *p* < .001), and more conduct problems (*β* = .149, *b* = .211, *p* = .008) as well as greater levels of hyperactivity/inattention (*β* = .123, *b* = .107, *p* = .026). In summary, higher sensation seeking was negatively related to internalising problem behaviour (especially emotional, but also peer-relationship problems) and showed positive associations with externalising problem behaviour (especially conduct problems, but also hyperactivity/inattention).

All statistical models presented in Table 3 were checked for interactions between each independent variable and age or gender. However, interactions did not reach significance and, consequently, were not included in the final models. Therefore, the associations reported in Table 3 can be assumed not to vary depending on child age or gender.

## Discussion

Whereas previous studies mainly investigated sensation seeking in older children, adolescents, and adults, nearly nothing is known about sensation seeking in preschool children. This study sought to fill this gap in the research by examining possible associations between sensation seeking and variables such as child age, gender, socio-economic status, and behavioural difficulties in 3- to 6-year-old children.

### Sensation seeking in the present sample

The questionnaire used in this study assessed novelty seeking, behavioural intensity, and thrill seeking in a sample of children. Our analyses suggest that these domains reflect different, but inter-related, facets of the same underlying trait of sensation seeking.

### Associations between sensation seeking and gender, age and SES

We observed a greater level of sensation seeking in boys than in girls, which was consistent with our hypothesis and supports findings for children, adolescents and adults in previous samples [10, 15, 21, 23]. However, we did not find significant gender differences in all sensation seeking domains. Specifically, boys were only found to score higher than girls for thrill seeking. Thrill seeking reflects the need for a variety of emotional challenges that raise excitement. Parents of children who scored highly on the TS scale state that their children are looking for excitement and thrills, such as would be provided by ‘Watch a video of a car exploding’, or ‘Go to a zoo where animals are not in cages’. Reasons for higher thrill seeking in boys vs. girls might lie in the ways they are socialised and educated. Parents interact differently with boys compared to girls and might, therefore, impact children’s behaviours and attitudes [47]. Another factor that might explain the gender difference is an underlying difference in courage. In a previous study, more courageous children were shown to exhibit more thrill- and adventure-seeking behaviour [48]. Novelty seeking reflects enthusiasm, a dislike of repetition and a preference for new experiences, and the ‘behavioural intensity’ scale assesses the desire to engage in activities involving speed or danger. For these facets of sensation seeking, no gender differences were shown. These findings suggests that preschool girls, like their male counterparts, feel the same need for new, varying and intense experiences.

In addition, our analyses revealed greater levels of sensation seeking in older children than in younger children, at least in the domains NS and TS. Even if the present study did not assess sensation seeking longitudinally, this outcome suggests that sensation seeking is a trait that develops early and has already increased by the time the child reaches preschool age. This result is in line with the suggestion that sensation seeking increases during childhood [16].

As expected, and as shown in previous studies in adults [12, 26], we found no significant association between children’s sensation seeking and their SES. This finding suggests that sensation seeking is evenly distributed across social classes and is independent of a family’s education, occupational status and income. Please note, however, that the study sample shows a trend towards a higher socioeconomic status. Therefore, this finding might not be generalisable to the general population.

### SSSYC and behavioural difficulties

We hypothesised that young children with a greater propensity for sensation seeking have more behavioural difficulties. In line with our expectations, we found positive relationships between sensation seeking and conduct problems. In particular, there is an association between behavioural intensity and thrill seeking, on the one side, and the likelihood of having more conduct problems, on the other. These results are in line with findings in children and adolescents [33, 34] and suggest that the link between sensation seeking and aggressive, quarrelsome behaviour and short-temperedness is already apparent in young childhood. In line with our hypothesis, we also found that children who present more thrill seeking showed more signs of hyperactivity/inattention.

Interestingly, our analyses revealed associations not only between sensation seeking and externalising behaviour, but also between sensation seeking and internalising behaviour. In adults, previous studies have yielded mixed results. In the present study, the analyses revealed a robust negative association between sensation seeking and emotional problems, which was observable in all domains of sensation seeking. Additionally, peer-relationship problems were found to relate to fewer indications of sensation seeking, at least in the domain of behavioural intensity. These findings suggest that higher levels of sensation seeking are negatively associated with internalizing behaviour. This suggestion is in line with previous studies in which lower sensation seeking was linked to symptoms of depression and anxiety [33, 49, 50]. However, sensation seeking has been shown to be related to other personality traits, e.g. extraversion and openness to experience [9]. These traits might mediate the observed relationship between sensation seeking and internalizing behaviour. Furthermore, due to their ‘courage’, children with higher sensation seeking might be especially popular in their peer group. This might be another explanation for the relationship between higher sensation seeking and fewer emotional and social problems. Future research should examine the mechanism underlying the associations between sensation seeking and early behavioural difficulties in children. Furthermore, it would be interesting to investigate the relationship in clinical samples, e.g. in children diagnosed with ADHD, behavioural disorders, or depression.

### Limitations

This is the first study to have examined sensation seeking in German three- to six-year-old children and investigated how this trait might be associated not only with socio-demographic and socio-economic parameters, but also with behavioural difficulties. However, certain limitations should be acknowledged. All measures were based on parental reports and, thus, the parents’ perceptions of their children’s behaviour. However, parents might not be the most reliable informants. Furthermore, the representativeness of the present sample was limited, especially with respect to the SES of participating families. As in other cohort studies, the household income and educational level of the study’s participants reflect a higher socioeconomic status than the general population [40, 51].

## Conclusion

The results of the present study indicate that boys are more sensation seeking than girls and suggest that sensation seeking increases with age. However, levels of sensation of seeking in children do not differ depending on the socio-economic status of their families. These results replicate findings in adults and strengthen the assumption that sensation seeking is a personality trait that is already observable in early childhood. Most interestingly, the findings show an association between a greater propensity for sensation seeking and externalising behavioural problems, whereas lower sensation seeking is associated with internalising behavioural problems.

## Abbreviations

BI: Behavioural intensity
M: Mean
N: Number
NS: Novelty seeking
SD: Standard deviation
SDQ: Strengths and Difficulties Questionnaire
SES: Socio-economic status
SSSYC: Sensation Seeking Scale for Young Children
TS: Thrill seeking
